# Bisphosphonate Associated Osteonecrosis of the Jaw: An Update on Pathophysiology, Risk Factors, and Treatment

**DOI:** 10.1155/2014/471035

**Published:** 2014-09-01

**Authors:** Lars Rasmusson, Jahan Abtahi

**Affiliations:** ^1^Department Oral and Maxillofacial Surgery, The Sahlgrenska Academy, University of Gothenburg, P.O. Box 450, 405 30 Gothenburg, Sweden; ^2^Maxillofacial Unit, Linköping University Hospital, 581 85 Linköping, Sweden

## Abstract

Osteonecrosis of the jaw in patients treated with bisphosphonates is a relatively rare but well known complication at maxillofacial units around the world. It has been speculated that the medication, especially long-term i.v. bisphosphonate treatment, could cause sterile necrosis of the jaws. The aim of this narrative review of the literature was to elaborate on the pathological mechanisms behind the condition and also to gather an update on incidence, risk factors, and treatment of bisphosphonate associated osteonecrosis of the jaw. In total, ninety-one articles were reviewed. All were published in internationally recognized journals with referee systems. We can conclude that necrotic lesions in the jaw seem to be following upon exposure of bone, for example, after tooth extractions, while other interventions like implant placement do not increase the risk of osteonecrosis. Since exposure to the bacterial environment in the oral cavity seems essential for the development of necrotic lesions, we believe that the condition is in fact chronic osteomyelitis and should be treated accordingly.

## 1. Introduction

The first report describing osteonecrosis of the jaw (ONJ) in patients receiving bisphosphonates came 2003 [1]. Since then this condition, sometimes called BRONJ (bisphosphonate-related osteonecrosis of the jaw), has shown increasing interest by dentists and oral-maxillofacial surgeons. It is defined as an area of exposed bone in the maxillofacial region that does not heal within 8 weeks in a patient who is currently receiving bisphosphonate medication and has not had radiation to the head-neck region. The diagnosis is usually made clinically. It is believed mainly to be associated with high dose intravenous bisphosphonate therapy, but sometimes the condition occurs also in patients with low-dose osteoporotic treatment. The current perception among dentists and oral-maxillofacial surgeons seems to be that low-dose bisphosphonate treatment for osteoporosis is linked to an increased incidence of ONJ, while on the other hand endocrinologists may suggest increased prescribing to decrease the incidence of osteoporotic fractures. This review aims to elaborate on the pathogenic mechanisms behind bisphosphate associated necrosis of the jaw and incidence, prevention, and treatment of the condition.

## 2. Methods

The present paper is authored as a narrative review contribution. Data synthesis and analysis: the articles were picked and sorted according to their corresponding key area of focus.

## 3. Results

Ninety-one studies were included, consisting of 9 reviews, 79 original papers, 2 letters and 1 thesis.

## 4. Discussion

### 4.1. Structure and Bioactivity of Bisphosphonates

Bisphosphonates (BPs) are antiresorptive drugs that act specifically on osteoclasts, thereby maintaining bone density and strength [2]. The drug is used for many indications including prevention and treatment of primary and secondary osteoporosis, hypercalcaemia, multiple myeloma, and osteolysis due to bone metastases and Paget's disease [3, 4]

**Figure 1:**
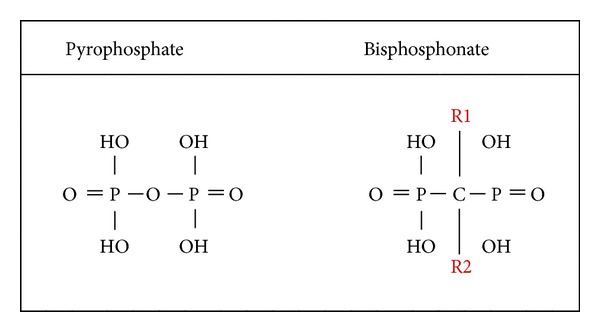
Chemical structure of pyrophosphate and bisphosphonate. R1 and R2 signify the side chains of bisphosphonate.

BPs act on both osteoblast and osteoclasts. It has been shown* in vitro* that BPs promote proliferation and differentiation of human osteoblast-like cells [5] and inhibit osteoclasts. The BPs are synthetic analogs with a P–C–P bond instead of the P–O–P bond of inorganic pyrophosphates, which are used as a bone-specific radionuclide in technetium 99 m methylene diphosphonate (Tc 99 m MDP) bone scans. Unlike pyrophosphates, bisphosphonates are resistant to breakdown by enzymatic hydrolysis, which explains their accumulation in the bone matrix and their extremely long half-life [6]. The P–C–P structure (Figure 1) allows a great number of possible variations, especially by changing the two lateral chains (R1 and R2) in the carbon atom. The two phosphate groups are essential for binding to the bone mineral such as hydroxyapatite and together with the R1 side chain they act as a “bone hook.” A hydroxyl (OH) group or amino group at the R1 position increases the affinity for calcium and thus for bone mineral [7, 8] Figure 1.

The structure and three-dimensional conformation of the R2 side chain determine the antiresorptive potency and the enhanced binding to hydroxyapatite [7, 9].

It is known that bisphosphonates containing a basic primary nitrogen atom in an alkyl chain such as alendronate are 10–100 times more potent at inhibiting bone resorption than earlier generation BPs like clodronate which lack this feature. Compounds that contain tertiary nitrogen such as ibandronate and olpadronate are even more potent at inhibiting bone resorption. Risedronate and zoledronate are among the most potent BPs, containing a nitrogen atom within a heterocyclic ring [10].

The gastrointestinal uptake of orally administrated BPs is low with a bioavailability of 0.3–0.7% [11, 12]. The poor absorption of BPs can probably be attributed to their very poor lipophilicity which prevents transcellular transport across epithelial barriers. Consequently BPs must be absorbed by the paracellular route, which means passage though the pores of tight junctions between the epithelial cells.

Bisphosphonates are completely ionized in blood at physiological pH (7.4). Therefore, plasma protein binding is high, expectedly as ion binding. Lin and coworkers [13] demonstrated that, in rats, alendronate binds to serum albumin and this binding seems to be dependent on serum calcium-levels and pH. Plasma protein binding in human has been found to be less with alendronate showing an unbound fraction 22% compared to 4% in rats [13].

Intravenous administration of a single dose of alendronate leads on the other hand to rapid accumulation of this drug in bone tissue, approximately 30% in 5 min and 60% in 1 hour [14]. The half-life in plasma is 1-2 hour and this rapid elimination is due to bone uptake and renal clearance. Once incorporated into the bone, bisphosphonates are liberated again only when the bone in which it was deposited is resorbed. Therefore the rate of the bone turnover influences the half-life of this drug [15].

The distribution of BPs in bone is determined by blood flow and favours deposition at sites of the skeleton undergoing active resorption [14].

Neither orally nor intravenously administrated BPs are metabolized in humans [16].

### 4.2. Mechanism of Action

During bone resorption, bisphosphonates impair the ability of the osteoclasts to form the ruffled border, to adhere to the bony surface and to produce the protons necessary for continued bone resorption [17–19].

Following cellular uptake, a characteristic morphological feature of bisphosphonate-treated osteoclasts is the lack of a ruffled border, leading to reduced adhesion to the bony surface. Bisphosphonates also promote osteoclast apoptosis by decreasing osteoclast progenitor development and recruitment [20]. Nevertheless, following exposure to certain bisphosphonates, inhibition of the osteoclast proton pumping H-ATPase phosphatases and lysosomal enzymes could also contribute to the loss of resorptive capacity of osteoclasts [21, 22].

Clodronates are the first generation, nonnitrogen-containing bisphosphonates which entered osteoclasts, incorporated into nonhydrolyzable analogues of adenosine triphosphate (ATP) and converted into methylene-containing (AppCp type) analogues of ATP. Accumulation of these toxic by-products interferes with mitochondrial function and ultimately leads to apoptosis of osteoclasts [23, 24].

In contrast, nitrogen-containing bisphosphonates (such as zoledronate and pamidronate) act by inhibiting farnesyl pyrophosphate (FPP) synthase and geranylgeranyl pyrophosphate (GGPP) synthase, two key enzymes in the mevalonate pathway. As a consequence, the disruption of the mevalonate pathway by nitrogen-containing bisphosphonates results in impaired protein prenylation and activation av small GTPases such as Ras, Rho, Rac, and Cdc42. The small GTPases are important signalling proteins regulating osteoclast morphology, cytoskeleton arrangement, membrane ruffling, and trafficking and cell survival [10, 25].

It has been suggested that another target of BPs could be the osteoblast, which in turn influence the osteoclasts. It has been shown experimentally that BPs inhibit the expression of receptor activator of NF-kappa B ligand (RANK-L) in rat osteoblast cells and increase the expression of osteoprotegerin (OPG) in human osteoblastic cells, suggesting that the antiresorptive effect of BPs is mediated by influence of osteoblasts on RANK-L signalling [26, 27].

### 4.3. Systemic and Local Delivery of Bisphosphonates

Several experimental studies showed that systemic bisphosphonates reduced alveolar bone loss [28–30]. In animal models, several investigators have shown that surface-immobilized bisphosphonates improve mechanical fixation of metal screws in terms of an increased bone-to-implant contact and pullout force [31–35]. Single systemic infusion of zoledronate has shown promising results on initial fixation of cementless orthopaedic implants [36, 37].

Local application of BPs during total joint surgery has been shown to reduce migration of metal prostheses as measured by radiostereometry [38].

In a recent series of randomized controlled trials, local treatment of periodontitis with a gel containing a very high concentration of alendronate was successful in regenerating a large part of lost bone, whereas placebo had little effect [39–41].

In the randomized study of 16 patients, a thin bisphosphonate-eluting fibrinogen coating improved the fixation of dental implants in human bone Abtahi et al. [42]. The efficacy of the topical administration of bisphosphonates in implant therapy has been investigated by Zuffetti et al. [43]. By the 5-year follow-up, no implant failure had been recorded in test group.

### 4.4. Osteonecrosis of the Jaw (ONJ)

**Figure 2:**
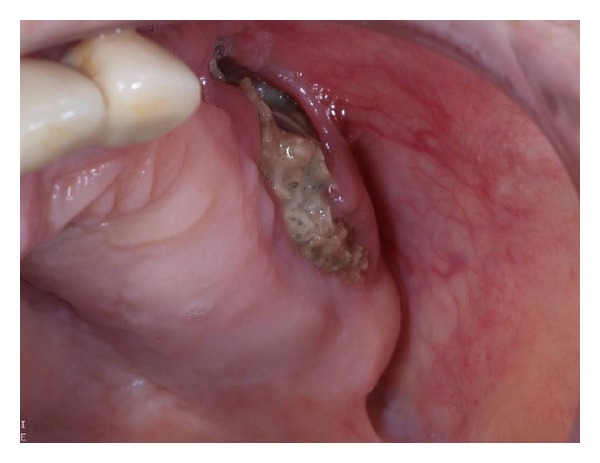
Exposed necrotic bone after tooth extractions in a patient treated with i.v. zoledronic acid.

Historically, osteonecrosis of the jaw (ONJ) was first reported by occupational exposure to white phosphorus which was called “*phossy jaw*” [44, 45]. ONJ has also seen in osteopetrosis, a rare inherited disease with impairment of bone resorption and remodeling [46]. More recently, ONJ is defined as a complication of head and neck radiotherapy [47]. The definition of ONJ is nonhealing exposed jawbone for more than 8 weeks in patients receiving BPs and without any local radiation therapy. Clinically, the disease presents as exposed alveolar bone that becomes evident following a surgical procedure such as tooth removal or periodontal therapy [48, 49] Figure 2.

Signs and symptoms that may occur before the development of clinically detectable osteonecrosis include pain, tooth mobility, mucosal swelling, erythema, and ulceration. The incidence of ONJ in bone malignancy cases, mainly treated with high dose intravenous bisphosphonates, is about 1–12% [48, 49].

Wang and coworkers [50] found that the incidence of ONJ was at least 3.8% in patients with multiple myeloma, 2.5% in breast cancer patients, and 2.9% in prostate cancer patients. In osteoporosis, bisphosphonate associated osteonecrosis of the jaw is rare and the incidence may not be greater than the natural background incidence. Epidemiological studies have indicated an estimated incidence of less than 1 cases per 100 000 person-years of exposure to oral bisphosphonates.

### 4.5. Pathogenesis

The etiology of ONJ remains uncertain. Initially, when the condition was called bisphosphonate-related osteonecrosis of the jaw (BRONJ) [48] its similarities with radiation-induced osteonecrosis led to the assumption that the condition started with sterile necrosis of the jaw bone. Therefore, the term osteonecrosis was used otherwise reserved for sterile bone death usually because of impaired blood supply. At that time, it was speculated that BPs could cause osteonecrosis through effects on blood vessels in bone, possibly by inhibition of vascular endothelial growth [51].

Later, it has been suggested that the condition does not begin as a form of classical osteonecrosis but in fact osteomyelitis from the start [52, 53].

Bacterial contamination with* Actinomyces* and* Staphylococcus* may play a role in maintaining osteomylitic wounds and because maxillofacial bone tissue containing BPs will resorb slowly, it is conceivable that contaminated bone cannot be removed fast enough to prevent the development of chronic osteomyelitis. This view is supported by the fact that similar lesions appear after treatment with anti-RANK-L antibodies that reduces osteoclast recruitment [54]. Thus, it appears that reduced resorptive activity is a key factor behind the impaired healing capacity of these lesions [55].

We suggest that the term BRONJ should be avoided and replaced by the term bisphosphonate associated osteomyelitis of the jaw, BAOJ, which better reflects the conditions aetiology.

Antibiotics can prevent the development of ONJ-like lesions in a rat model [56]. One hundred twenty animals underwent tooth extraction and received combination of dexamethasone and pamidronate during different time periods. Animals which received the same treatment except for the addition of penicillin showed four times less ONJ-like lesions than the other group. There is no clinical study on the use of antibiotics associated with ONJ. However, in the clinical situation antibiotics has its use since the condition is considered osteomyelitis of the jaw.

The antiangiogenic role of bisphosphonate is still unclear and ONJ proceeds despite the use of antibiotics in some cases. One explanation could be the fact that bacterial contamination maintains chronic osteomyelitis of the jaws. Another explanation is perhaps the reduced microcirculation of the gingiva causing the soft tissue unable to heal.

Corticosteroids and chemotherapeutics have been suggested as factors that can predispose to ONJ or increase the risk of developing ONJ; the duration of BP therapy also appears to be related to the likelihood of developing necrosis with longer treatment regimens associated with a greater risk [55]. The time to develop osteonecrosis after i.v. zoledronate treatment was in mean 1.8 years, after i.v pamidronate 2.8 years and after oral BP therapy, like alendronate, the mean time was 4.6 years [57].

Numerous studies have explored the toxic effect of BPs on a variety of epithelial cells [58–62]. There is clear documentation of bisphosphonate toxicity to gastrointestinal epithelia [63]. It has been suggested that high concentrations of bisphosphonate in the oral cavity (bone tissue) disrupt the oral mucosa [64]. Failure of healing of the soft tissue may cause secondary infection of the underlying bone. However, this theory has not yet been accepted by investigators. Recently, in a rat model of ONJ, following tooth extraction a high dose of alendronate (200 *μ*g/kg) did not cause ONJ-like lesions [65]. When calculated as dose per body weight per day, the rat dose was 100 times higher than the human dose.

### 4.6. Clinical Characteristics

Blood supply to the cortical bone is derived from the periosteum and exposed bone surface is indicating necrosis in the underlying bone layers. The condition can then progress into a more severe bony lesion with nerve disturbances, mobile teeth, fistulas, and in the end fracture [66]. Pain is common and these signs and symptoms are often evident in patients with jaw bone osteomyelitis that are not on BP treatment. Radiographs may show sclerotic bone, sclerotic lamina dura around individual teeth, and widened periodontal ligaments but there are no report published indicating specific features for BP associated osteomyelitis [67].

### 4.7. Incidence

The incidence of BP associated osteomyelitis can be divided into 2 groups: the high dose i.v treated cancer patients and osteoporotic patients. In a systematic review, Kahn et al. found that, for the first group, the cumulative incidence varied from 1% to 12% after 36 months of treatment [66]. However, most of the reported cases have been related to intravenous use of bisphosphonates (zoledronic and pamidronic acid) to control metastatic bone disease or multiple myeloma. The incidence of ONJ in these studies ranges from 4 to 10% [1, 68, 69] and the mean time of onset varies from 1 to 3 years [55, 70, 71].

Osteoporosis is a common and costly condition that impaired quality of life [71]. It is estimated that 10 million individuals (aged >50 years) in the United States have osteoporosis, by 2010 [72]. Few studies have reported the prevalence of ONJ in persons receiving exclusive oral bisphosphonate therapy. No cases of ONJ were reported by Felsenberg et al. among clinical trials involving almost 17000 patients [73]. The authors estimated the worldwide reporting rate of ONJ to be <3/100,000 years of exposure [72]. In osteoporosis patients, by systemic review Kahn et al. estimated incidence of ONJ to be <1 case per 100,000 person-years of exposure [66]. Similar findings have been reported by German investigators, as determined by cases captured by a German Central Registry [73, 74]. By using postmarketing surveillance method Abtahi et al. identified one case of ONJ among 952 patients, who had received chronic oral bisphosphonate therapy [75]. Moreover, these findings contrast to those from an Australian study, which identified ONJ cases by nationwide maxillofacial surgeon survey [70].

The trigger for developing necrotic bone in BP treated patients seems to be dental extractions. A review of 114 cases of BP associated ONJ in Australia showed that 73% of the cases occurred after dental extractions. The frequency of ONJ in BP treated osteoporotic patients was 0.01%–0.04% and if dental extraction occurred 0.09%–0.34%. In patients on BPs for bone malignancies, the incidence was 0.33%–1.15% and after dental extractions 6.7%–9.1% [70].

### 4.8. Risk Factors

There are general and local risk factors for development of ONJ.

*General risk factors* include malignancies, chemotherapy, glucocorticoid treatment, and high dose or long-term bisphosphonate treatment [48, 66].

**Figure 3:**
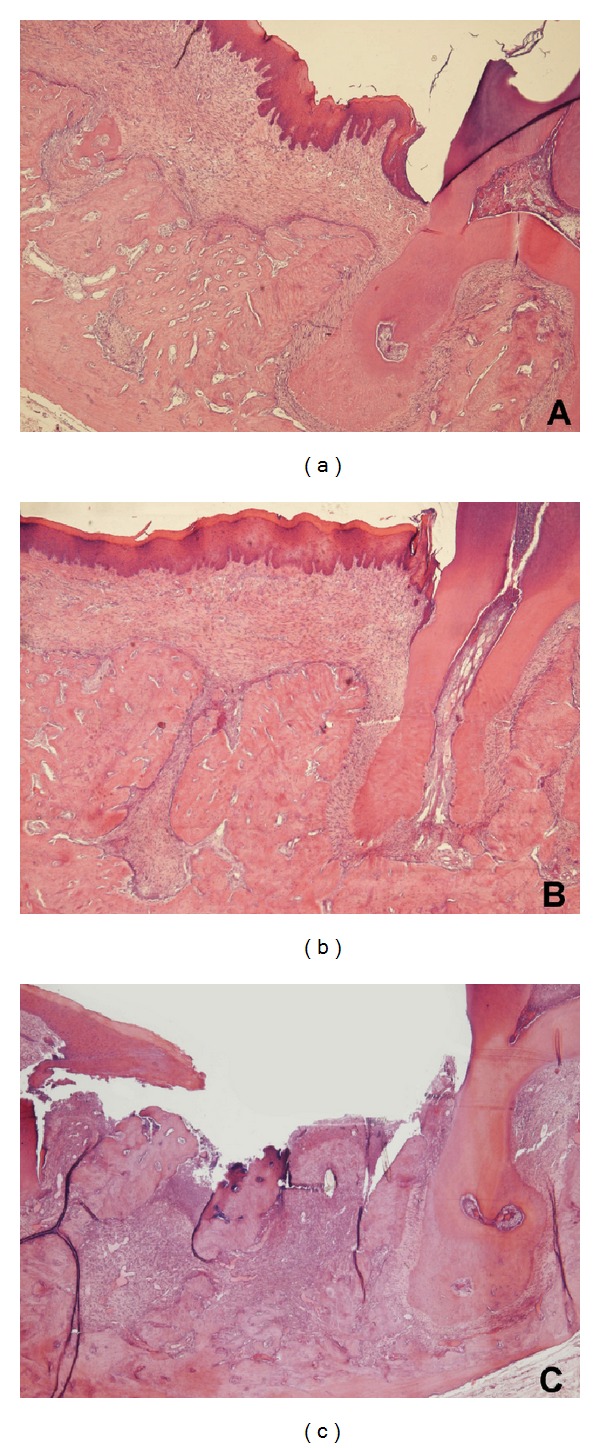
Histological sections showing the region of the second molar 14 days after extraction in male Sprague-Dawley rat. (a) Control rat with no treatment, (b) BP treated with coverage, and (c) BP treated without coverage. Note necrotic tissue.

*Local risk factors* include anatomical features where protruding cortical bone with thin mucosal coverage like tori and exostoses implies greater risk for necrosis as well as periodontal disease, any surgical intervention which breaks the mucosal lining, especially tooth extractions [48, 67]. In an experimental study by Abtahi and coworkers [75], it was shown that immediate soft tissue coverage after tooth extraction prevented ONJ completely whilst all noncovered sites developed ONJ in osteoporotic rats treated with alendronate, Figure 3.

The use of bisphosphonates is associated with the development of ONJ in some patients. Length of exposure seems to be the most important risk factor for this complication with an estimated range from 1.6 to 4.7 years, depending on BPs type [55]. Subsequent to ONJ development the minimum duration of use was reported to be 6 months [76, 77]. Barasch and coworkers showed that the risk for development of ONJ begins within 2 years of treatment, for both cancer and noncancer patients, showing that even the less potent bisphosphonates are linked to ONJ after a relatively brief treatment period [76]. Furthermore, for noncancer patients this risk seems to increase substantially after 5 years. This highlights the importance of drug holiday after 5 years of treatment. In a prospective study by Bamias et al. the incidence of ONJ was studied among patients treated with bisphosphonates for bone metastases. The incidence of ONJ increased with time to exposure from 1.5% among patients treated for 4 to 12 months to 7.7% for treatment for 37 to 48 months [77].

### 4.9. Bisphosphonates and Oral Implant Therapy

In a systematic review from 2009, Madrid and Sanz [78] included studies where patients had been on BP treatment for 1–4 years before implant placement. None of the patients developed osteonecrosis up to 36 months postoperatively and the implant survival rate ranged from 95 to 100%. This may indicate that exposed/noncovered bone is necessary for bacterial invasion and an osteomyelitic process.

Furthermore, in a study from 2010, Koka and coworkers found high implant survival rates for both bisphosphonate users and nonusers in postmenopausal women [79].

### 4.10. Treatment

The optimal treatment strategy for ONJ is still to be established. Cessation of BP treatment will not be sufficient. A multidisciplinary team approach for evaluation and management of the conditions is recommended including a dentist, an oral-maxillofacial surgeon, and an oncologist. In early stages, surgical debridement and coverage has been successful [80]. Hyperbaric oxygen (HBO) is an effective adjunctive therapy in situations in which normal wound healing is impaired and the effects of HBO therapy have been discussed by several investigators [81, 82]. The authors showed that patients with ONJ, adjunctive HBO_2_ therapy had remission or improvement in over 62.5% of patients. Laser therapy at low intensity has been reported for treatment of ONJ by improving reparative process, increasing osteoblastic index, and stimulating lymphatic and blood capillaries growth [83–85].

Segmental osteotomies are recommended only for severe cases [86–89], due to relatively high levels of morbidity and impaired quality of life for the patients [90].

In a study by Holzinger et al. [91], 108 patients with bisphosphonate therapy underwent surgery and 88 patients were followed for a mean period of 337 days. Surgical treatment improved the stage distribution from 19% stage I, 56% stage II, and 25% stage III to 59% intact mucosa, 19% stage I and 13% stage II and 8% stage III. The improvement in the stage of disease achieved by surgery was statistically significant. However, the choice between surgery and conservative therapy is a difficult issue and must be made on an individual basis.

Recently there have been discussions regarding the applicability of “drug holidays” to minimize long-term bisphosphonate exposure and avoid potential adverse events such as ONJ. However, given the long half-life of bisphosphonates in bone (measured in years) whether or not temporary cessation of treatment with these agents would reduce associated risks is not known. These questions require further study.

Antibiotics: Samples should be taken for culture and sensitivity testing before starting ab treatment. Traditionally, the antibiotics of choice to treat osteomyelitis will include Flucloxacillin or Clindamycin.

Prevention is a cornerstone to reduce the incidence of ONJ and before starting BP therapy, the patient should be referred for thorough dental evaluation to identify and treat any potential source of infection. Start of BP therapy should be delayed by 4–6 weeks to allow appropriate bone healing [90].

The treatment of bisphosphonate-related osteonecrosis of the jaw is generally difficult. For this reason, prevention plays a predominant role in the management of this condition.

## 5. Conclusion

The present narrative review, based on experimental and clinical original papers as well as previous reviews, indicates that osteonecrosis of the jaw in BP treated patients seems to be triggered by exposed bone and subsequent bacterial contamination, typically after dental extraction, and that sterile necrosis of the jaw is unlikely. We therefor suggest that the condition could be coined “*Bisphosphonate associated osteomyelitis of the jaw.*”
